# Brown Adipose Tissue and Novel Management Strategies for Polycystic Ovary Syndrome Therapy

**DOI:** 10.3389/fendo.2022.847249

**Published:** 2022-05-19

**Authors:** Qiaoli Zhang, Rongcai Ye, Yuan-Yuan Zhang, Chen-Chen Fan, Jun Wang, Shuyu Wang, Suwen Chen, Xiaowei Liu

**Affiliations:** ^1^Department of Human Reproductive Medicine, Beijing Obstetrics and Gynecology Hospital, Beijing Maternal and Child Health Care Hospital, Capital Medical University, Beijing, China; ^2^Key Laboratory of Animal Ecology and Conservation Biology, Institute of Zoology, Chinese Academy of Sciences, Beijing, China; ^3^Department of Reproductive Regulation (Family Planning), Beijing Obstetrics and Gynecology Hospital, Beijing Maternal and Child Health Care Hospital, Capital Medical University, Beijing, China; ^4^Department of Obstetrics, Beijing Obstetrics and Gynecology Hospital, Beijing Maternal and Child Health Care Hospital, Capital Medical University, Beijing, China

**Keywords:** brown adipose tissue (BAT), adipokine, activation, polycystic ovary syndrome (PCOS), metabolic disorder

## Abstract

Brown adipose tissue (BAT), a unique tissue, plays a key role in metabolism and energy expenditure through adaptive nonshivering thermogenesis. It has recently become a therapeutic target in the treatment of obesity and metabolic diseases. The thermogenic effect of BAT occurs through uncoupling protein-1 by uncoupling adenosine triphosphate (ATP) synthesis from energy substrate oxidation. The review discusses the recent developments and progress associated with the biology, function, and activation of BAT, with a focus on its therapeutic potential for the treatment of polycystic ovary syndrome (PCOS). The endocrine activity of brown adipocytes affects the energy balance and homeostasis of glucose and lipids, thereby affecting the association of BAT activity and the metabolic profile. PCOS is a complex reproductive and metabolic disorder of reproductive-age women. Functional abnormalities of adipose tissue (AT) have been reported in patients with PCOS. Numerous studies have shown that BAT could regulate the features of PCOS and that increases in BAT mass or activity were effective in the treatment of PCOS through approaches including cold stimulation, BAT transplantation and compound activation in various animal models. Therefore, BAT may be used as a novel management strategy for the patients with PCOS to improve women’s health clinically. It is highly important to identify key brown adipokines for the discovery and development of novel candidates to establish an efficacious therapeutic strategy for patients with PCOS in the future.

## Introduction

Adipose tissues (ATs), including brown (BAT), white (WAT), and beige (BeAT) tissues, perform essential functions in the maintenance of whole-body energy homeostasis (1). BAT is a specialized fat tissue that serves as the primary site for adaptive nonshivering thermogenesis to generate heat under cold stress in mammals. BAT participates in primary metabolism and energy expenditure (EE), and it can be quickly stimulated by thermal or dietary stimulation (2). A recent study showed that an increase in BAT mass and/or function could be an effective therapeutic target for the treatment of obesity and other related metabolic diseases in patients (3).

**Figure 1:**
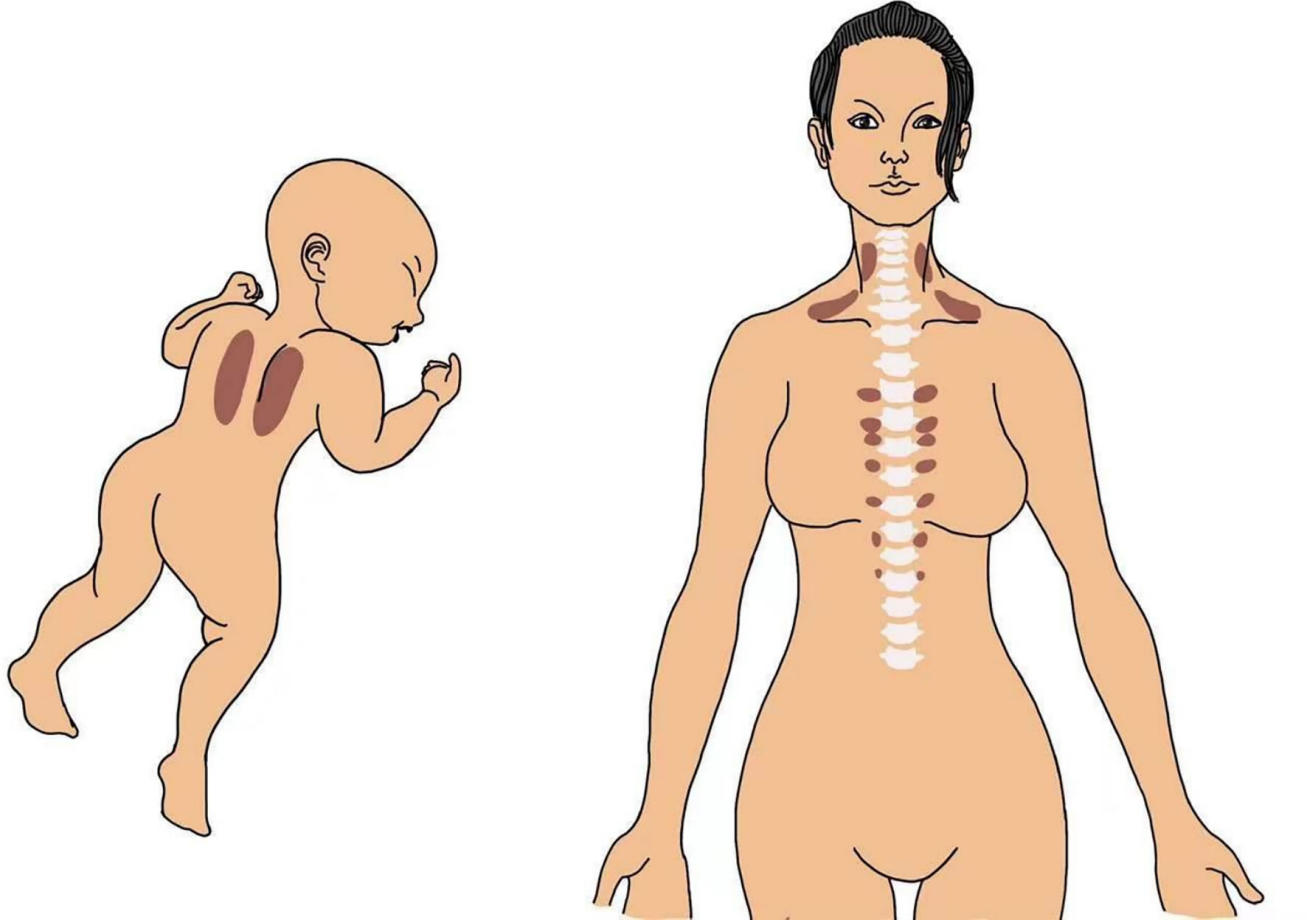
A drawing showing representative BAT occurrence in the interscapular region in an infant and in the supraclavicular, cervical, and paravertebral regions in an adult human.

BAT depots in the interscapular region of infants have been clearly visualized by magnetic resonance imaging (MRI), and BAT could be detected and quantified in the supraclavicular, cervical, and paravertebral regions of adults by positron emission tomography–computer tomography (PET-CT) with 18F-fluorodeoxyglucose (FDG) (4–7) (**Figure 1**). PET-CT imaging revealed a strong positive correlation between BAT activity and the basal metabolic rate. In addition, the level of BAT activation is inversely correlated with age, body mass index (BMI) (7, 8), and adiposity in adults. The young lean and females possess higher metabolically active BAT (9).

Brown adipocytes have unique features, such as multilocular lipid droplets, upregulated uncoupling protein-1 (UCP1), rich mitochondria and capillarity (3). The thermogenesis of BAT is largely dependent on UCP1 (a marker of BAT) on the mitochondrial inner membrane for energy dissipation (10, 11). UCP1 “uncouples” adenosine triphosphate (ATP) synthesis from the oxidation of energy substrates, thereby promoting nonproductive EE through increased mitochondrial uncoupling (5, 9, 10). The activity and development of brown adipocytes are regulated by the sympathetic nervous system (SNS). Thermogenesis mediated by the SNS is highly regulated by neurons in the hypothalamus and brainstem, and brown adipocyte thermogenesis is controlled by the leptin-melanocortin pathway (12, 13).

## BAT-Secreted Factors — Adipokines Improve Metabolic Health

### Brown Adipokines

**Figure 2:**
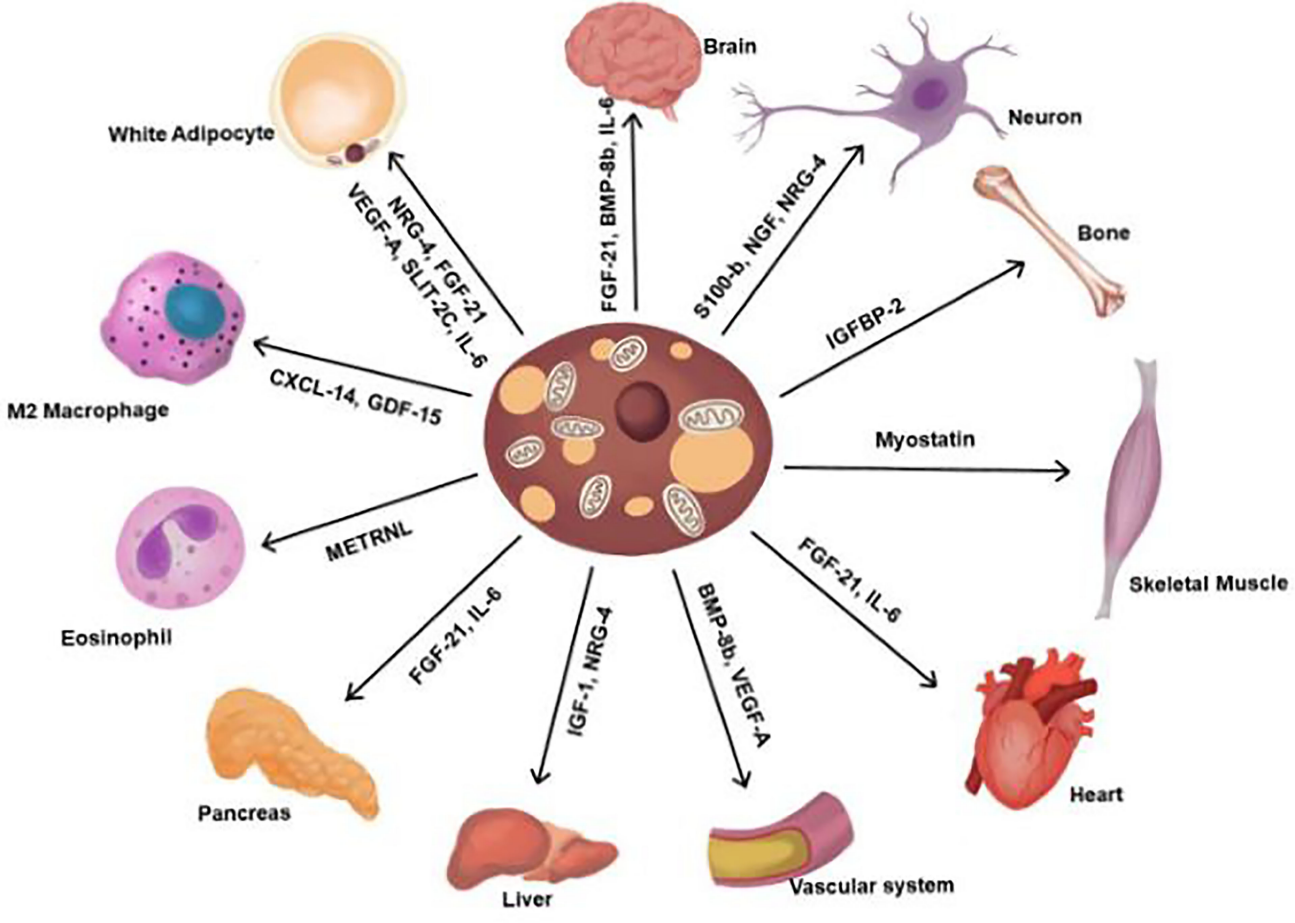
Adipokines secreted from BAT. Contribute to the regulation of various functions. FGF-21, fibroblast browth factor 21; BMP-8b, bone morphogenetic protein 8b; IL-6, interlukin-6; NGF, nerve growth factor; NRG-4, neuregulin 4; IGFBP2, insulin-like growth factor-binding protein 2; VEGF-A, vascular endothelial growth factor A; IGF-1, insulin growth factor-1, METRNL, meteorin-Like; CXCL14, chemokine (C-X-C motif) ligand 14; GDF-15, Growth and differentiation factor 15; SLIT2-C, C-terminal fragment of SLIT-2.

Brown adipokines are regulatory factors secreted by brown adipocytes that posses autocrine, paracrine, and endocrine activities and regulate BAT differentiation (14). Some adipokines display hormonal functions that increase BAT activity, improve the metabolic profile of glucose and lipid homeostasis, and mediate the browning of WAT (15–17). Moreover, thermogenic stimuli induce brown adipocytes to secrete signalling molecules targeting the sympathetic nerve, vasculature, and immune cells for tissue remodelling. Adipokines also activate distant organs and cells, to execute various local and systemic functions (**Figure 2**) (14, 18).

BAT can release signalling peptides, lipokines, and exosomal miRNAs to regulate metabolism in distant tissues to coordinate the metabolic function of the whole body (19, 20). BAT contributes to metabolic homeostasis *via* UCP1-mediated thermogenesis and secretory cytokines such as adiponectin and fibroblast growth factor 21 (FGF-21) (21). A total of 101 proteins were found from the secretome of brown adipocytes, and ependymin-related protein 1 (EPDR1) functions in brown adipocyte development (22). The thermogenesis of BAT could significantly impact the long-term regulation of energy homeostasis and body weight; therefore, an increase in BAT volume and/or activity may become a novel therapeutic strategy in the treatment of patients with obesity and metabolic diseases.

### Therapeutic Potential of BAT

BAT is a powerful sink to drain and oxidize glucose and triglycerides from the blood; therefore, it has therapeutic potential for the treatment of various metabolic diseases through its anti-glycaemic, anti-lipidaemic, and anti-obesity effects (4, 23). Brown fat can produce 300-fold more heat per unit mass than any other organ in the body after maximal stimulation, accounting for approximately 10% of the total daily heat (24). One of the major focuses of BAT-related pharmacological research is the treatment of obesity resulting from a prolonged energy imbalance due to calorie consumption exceeding calorie expenditure, consequently leading to a range of metabolic diseases, including hyperglycaemia, type 2 diabetes mellitus (T2DM), and hyperlipidaemia (25, 26).

Vascular lipoprotein homeostasis is regulated by BAT *via* enhancing triglyceride-rich lipoprotein (TRL) turnover and transporting lipids into BAT. Thus, high triglyceride levels may be effectively reduced by the activation of BAT (27). BAT has therapeutic potential in the treatment of cardiovascular diseases because of its effects on increasing fatty acid catabolism and reducing plasma triglycerides, atherosclerosis, and inflammation in obese patients (28). In addition to the potential effects of stimulating BAT, beige adipocytes secrete insulin-like growth factor-binding protein 2 (IGFBP-2), which has an anabolic effect on bone tissues, so it may be effective for the treatment of skeletal deformities (28).

Taken together, human thermogenic adipocytes could serve as therapeutic targets by three different approaches: 1) an increase in BAT mass by inducing BAT progenitors; 2) an increase in WAT browning by enhancing the formation of beige adipocytes; and 3) an increase in BAT function through upregulating the regulatory pathways of BAT (29).

### “Browning” of WAT

BAT and BeAT are the primary sites for adaptive nonshivering thermogenesis and are vital for metabolic regulation through the secretion of adipokines in response to different pathophysiological stimuli. Classical brown adipocytes are localized in developmentally programmed BAT depots both in rodents and human infants. “Beige” (from “brown-in-white”) adipocytes are brown adipocytes that, in response to thermogenic stimuli such as chronic cold exposure, undergo the “browning” process in white fat and are important components of BAT depots in adults and may become a novel therapeutic target for the treatment of obesity, insulin resistance (IR), and T2DM (30, 31). No evidence has shown that the thermogenic function and mechanisms are different between beige and brown adipocytes (32). WAT contains a single lipid droplet, fewer mitochondria, and UCP1 negative (33). WAT is responsible for storing fat as energy. It stores energy in the form of triglycerides and causes obesity and multiple metabolic diseases (34). It also releases energy into the body in the form of free fatty acids and glycerol (35). WAT, including subcutaneous fat and visceral fat, can undergo browning. However, the subcutaneous fat (e.g., inguinal) is particularly prone to browning and exhibited significantly increased levels of UCP1. The browning of WAT, as a therapeutic strategy, commonly refers to the process of long-term therapy with peroxisome proliferator-activated receptor (PPAR) agonists and increases the formation of beige adipocytes (30).

### Activation of BAT

The SNS controls the activity of BAT, and BAT is activated by metabolic and hormonal signals (11, 36). Activation of brown and/or beige fats increases EE and decreases hyperglycaemia and hyperlipidaemia. The activation of BAT/BeAT leads to increasing lipolysis and inhibits the processes of autophagy and mitophagy (37).

#### Cold Exposure

Prolonged or chronic exposure to cold could recruit and activate BAT with an EE increase and rapid lipid and glucose oxidation. Studies indicate that cold exposure stimulates the expression of UCP1 and that the effect is greater in women than in men (7). BAT becomes activated by cold exposure in the extrauterine environment and by strong endocrine stimulation at birth (2, 38). In contrast, acute and repeated exposure to mild cold (17-19°C) could increase the volume and activity of BAT in adults. These effects are mediated by the SNS and transient receptor potential (TRP) channels.

#### Diet-Induced Thermogenesis (DIT)

In addition to cold exposure, meal intake, particularly with food rich in protein and chemicals such as capsinoids, could induce BAT thermogenesis, namely DIT, and represents a relatively large component of daily total EE (39). TRP members frequently serve as chemical receptors for plant and food metabolites; for example, TRP vanilloid 1 (TRPV1) agonists, including capsaicin and capsinoids, could mimic the effects of cold exposure on the reduction in body fat by activating and recruiting BAT. The antiobesity effect of food ingredients, including catechins, in tea may occur through the TRP-SNS-BAT activation axis (40, 41).

#### Other Channels

In addition to DIT, the activity of BAT could be enhanced by other factors *via* both central and peripheral actions. For example, thyroid hormone could activate BAT centrally through binding to thyroid receptors in brown adipocytes to directly induce the expression of thermogenic genes. The neurotransmitter orexin can enhance the function of BAT by modulating sympathetic outflow and inducing the differentiation of brown fat precursors (42). β-Adrenergic agents can activate BAT thermogenesis and induce the browning of WAT (43). Thiazolidinedione, a PPAR gamma (PPARγ) activator, could induce WAT browning by recruiting existing BAT depots (44–46), but its effect depends on the concurrent activation of noradrenergic signals by effective thermogenic induction (47). Studies have shown that both irisin and melatonin can activate BAT and that transplantation of brown adipocyte stem cells may increase thermogenesis from both brown and beige adipocytes (48). Ginseng extract (GE) can activate BAT and enhance energy metabolism (49). Metformin may improve UCP1 and mitochondrial biogenesis in the BAT, however, it’s ineffective for body mass (50). The differentiation of brown adipocytes requires several receptors/transcription factors, including PPARγ, PPARγ-coactivator-1alpha (PGC1alpha), PRD1-BF1-RIZ1 homologous domain-containing 16 (PRDM16), CCAAT/enhancer-binding protein beta (C/EBP-beta), and bone morphogenetic protein 7 (BMP7), to facilitate the acquisition of the thermogenic phenotype of BAT, which is ultimately mediated by UCP1 (6). BAT could also be activated by the natriuretic peptides FGF-21 and BMP8b (42).

## BAT as a Novel Management Strategy For Polycystic Ovary Syndrome (PCOS) Therapy

### PCOS

PCOS is a serious medical condition associated with defects in metabolic, reproductive and psychological functions, affecting approximately 5-20% of reproductive-age women (51, 52). It manifests as a heterozygous entity of menstrual cycle abnormalities, anovulation, IR, hirsutism and androgenetic alopecia (53). Hyperinsulinemia and IR play an important role in the pathophysiology and metabolic manifestations of PCOS (54). Epidemiological studies have revealed that 38-88% of women with PCOS have central adiposity, overweight or obesity (55). PCOS women with androgen excess and IR are prone to visceral fat hypertrophy, and early-onset impairment of glucose tolerance is present in 30-40%, T2DM in 10%, borderline or high lipid levels in 70% and metabolic syndrome (MS) in 50% (56). Therefore, PCOS is closely related to metabolic disturbance and is considered a metabolic disorder (57).

Furthermore, PCOS is linked to impaired AT physiology and presents a greater risk of non-alcoholic fatty liver disease (NAFLD) (58–61). Women with PCOS are more vulnerable to endothelial dysfunction, atherosclerosis, and cardiovascular diseases (CVDs), with a 2.7-fold increased risk of developing endometrial carcinoma (62). Moreover, PCOS may increase the risk of depressive, anxiety, and sleep disorders and reduce quality of life (QoL), particularly in patients with hirsutism, weight gain and acne (63). PCOS patients with infertility present worse QoL because of psychological and emotional distress (64).

### General Management of PCOS

PCOS has significant clinical manifestations, including diverse metabolic, reproductive and psychological features (65). Recently, the new International Evidence-based Guideline for the Assessment and Management of PCOS highlighted the importance of lifestyle interventions, including diet, exercise and behaviour, as the first-line management to improve the signs and symptoms of PCOS (66, 67). Even just 5% of body weight loss could meaningfully improve insulin sensitivity, hyperandrogenism, menstrual irregularity, and other reproductive and metabolic features clinically (68, 69).

In addition, the management of hyperandrogenism and/or irregular menstrual cycles in patients with PCOS should be recommended with combined oral contraceptives (COCPs) (54). Metformin combined with lifestyle changes could improve weight, hormonal and metabolic outcomes with greater benefit achieved in patients with diabetes risk factors and impaired glucose tolerance (70). In addition to lifestyle intervention, anti-obesity medications may be used for obese patients with PCOS (51). Therefore, metformin is recommended alone or in combination for PCOS therapy, primarily for metabolic conditions. Currently, inositol, an experimental therapy, may be considered for PCOS (71, 72). Statins are safe and effective for treating dyslipidaemia in patients with PCOS (73). For the treatment of PCOS women with anovulatory infertility, aromatase inhibitors such as letrozole are the recommended first-line therapy, with clomiphene and metformin alone or in combination. Gonadotrophins are a second-line therapy (53, 74, 75).

The benefit of lifestyle modification, as first-line management, highly depends on the self-efficacy of patients, and the results were not consistent with existing evidence (76). It must be acknowledged that no single agent or management is effective in treating all metabolic disorders in PCOS patients (77). Although insulin-sensitizing agents, including metformin, have been used for the treatment of PCOS patients with metabolic aspects, the efficacy is limited for the reduction in weight and cardiovascular risk (77).

### BAT for PCOS Therapy

Studies have shown that BAT activity was decreased in patients with PCOS and in a rat model of PCOS, possibly due to increased central adiposity (78) and the main manifestations of IR and inflammation (79). AT dysfunction promotes metabolic disorders in the peripheral tissues of PCOS patients with larger adipocytes, lower activity of lipoprotein lipolytic enzyme, and impaired capacity of catecholamine-mediated lipolysis (80). Furthermore, decreased EE may be related to hypofunction of BAT in female mice with PCOS (81). Therefore, the activation of BAT is a potential therapeutic option for the treatment of PCOS to reverse metabolic disorders (68, 82, 83).

### Cold Exposure

Cold exposure of BAT can increase EE and lead to body weight loss (84). Cold-stimulated BAT activity is common in human adults, with a prevalence ranging from 30% to 100% depending on cohort studies (84). BAT activity could be increased by decreasing the ambient temperature or by planned cold exposure in human dwellings, which may further decrease body fat (11). However, the health benefits of cold exposure were inconsistent in patients and animals (85, 86). A recent study showed that cold exposure to 4°C for 20 days reversed the acyclicity of the oestrous cycle and reduced the circulating levels of testosterone and luteinizing hormone (LH) by activating endogenous BAT in rats with PCOS (87). Furthermore, the expression of steroidogenic enzymes and inflammatory factors was significantly reduced in the ovaries of rats with PCOS. Histological analysis showed that cold exposure significantly improved ovulation and fertility with a reduction in cystic ovarian follicles and an increase in the corpus luteum in rats with PCOS (87). These findings indicate that cold exposure may be a novel strategy for the treatment of PCOS.

### BAT Transplantation

BAT transplantation could normalize glucose tolerance and reduce tissue inflammation and diabetes markers of polyuria, polydipsia, and polyphagia, leading to euglycaemia. These effects are insulin independent but correlate with BAT recovery in mice (88). BAT transplantation also significantly increased the levels of adiponectin and leptin in mice (88). BAT transplantation was effective in improving energy metabolism and insulin sensitivity, preventing weight gain induced by a high-fat diet (HFD), and reversing pre-existing obesity in mice (89). BAT transplantation significantly improved IR and liver steatosis and reduced body weight gain with increased oxygen consumption and decreased total body fat mass in Ob/Ob mice (90).

The recovery of BAT activity could improve PCOS, and multiple studies have shown that BAT transplantation reversed polycystic ovaries, improved IR and infertility in rats and mice with PCOS (91, 92). BAT transplantation could also significantly enhance endogenous BAT activity and increase the level of circulating adiponectin and insulin sensitivity, thereby eventually ameliorating hyperandrogenism, acyclicity polycystic ovaries and infertility in rats with PCOS (92). In addition, BAT transplantation dramatically rescued PCOS phenotypes, which is consistent with the reported result of adiponectin protein administration (92). A recent study demonstrated that xenotransplantation of rat BAT could significantly recover ovarian function and fertility in PCOS mice (91).

### Activation of Endogenous BAT to Enhance BAT Activity

BAT activation by long-term cold exposure and BAT transplantation does not seem to apply to most patients with PCOS clinically. Therefore, the activation of endogenous BAT with natural compounds could be an effective novel therapeutic approach for the treatment of patients with PCOS. It has been reported that treatment with rutin (a flavonoid) for three weeks could increase BAT activation and improve thermogenesis and insulin sensitivity in rats with PCOS (93). Additionally, the expression of ovarian steroidogenic enzymes was upregulated, including steroid 17 alpha-hydroxylase/17,20 lyase (P450C17), aromatase, 3β-hydroxysteroid dehydrogenase (3-HSD), 17β-hydroxysteroid dehydrogenase (17-HSD) and steroidogenic acute regulatory protein (STAR). Moreover, treatment with rutin normalized acyclicity and the serum level of LH, and a large number of mature ovulated follicles were observed with a reduction in cyst formation in rats with PCOS (93). An additional study showed that rutin could enhance the activity of BAT and induce the formation of beige adipocytes, thereby ameliorating obesity and IR in obese mice (94). Great efforts have been made to find effective compounds that can activate BAT for the treatment of patients with PCOS.

## Conclusions

This review highlights the recent developments and progress in the biology and pharmacological therapy of BAT for the treatment of PCOS. We also discuss the thermogenic potential of BAT for the prevention and treatment of obesity and metabolic diseases. It is highly important to identify the major brown adipokines and their roles to discover novel candidates and effective therapeutic strategies for the treatment of PCOS. BAT has therapeutic potential as a “metabolic panacea” for anti-glycaemic, anti-lipidaemic and weight loss effects in the whole body.

PCOS is a complex reproductive and metabolic endocrinopathy of women and the main cause of infertility with various clinical manifestations. Lifestyle management and pharmacological interventions are helpful, but the effectiveness is not consistent, and they do not completely meet the needs of patients with PCOS. Numerous studies have shown that BAT activity is decreased in patients with PCOS, and an increase in the mass and/or activity of BAT may be effective and could provide a novel therapeutic approach for the treatment of PCOS, such as cold stimulation, BAT transplantation and drug activation. BAT may be effective in reversing metabolic morbidities and inducing weight loss and could become a novel promising therapy for the treatment of PCOS. However, further extensive research is required to find the possible molecular mechanism preclinically and to validate its significance in women with PCOS clinically.

## Author Contributions

QZ conceptualized and wrote the manuscript. RY edited the manuscript. YZ, CF, and JW consulted literature and participated in the discussion. SC, XL and SW revised and approved the final version. All authors contributed to the article and approved the submitted version.

## Conflict of Interest

The authors declare that the research was conducted in the absence of any commercial or financial relationships that could be construed as a potential conflict of interest.

## Publisher’s Note

All claims expressed in this article are solely those of the authors and do not necessarily represent those of their affiliated organizations, or those of the publisher, the editors and the reviewers. Any product that may be evaluated in this article, or claim that may be made by its manufacturer, is not guaranteed or endorsed by the publisher.
